# Cell-Type Independent MYC Target Genes Reveal a Primordial Signature Involved in Biomass Accumulation

**DOI:** 10.1371/journal.pone.0026057

**Published:** 2011-10-19

**Authors:** Hongkai Ji, George Wu, Xiangcan Zhan, Alexandra Nolan, Cheryl Koh, Angelo De Marzo, Hoang Mai Doan, Jinshui Fan, Christopher Cheadle, Mohammad Fallahi, John L. Cleveland, Chi V. Dang, Karen I. Zeller

**Affiliations:** 1 Department of Biostatistics, Johns Hopkins Bloomberg School of Public Health, Baltimore, Maryland, United States of America; 2 Department of Gynecology and Obstetrics, Johns Hopkins University School of Medicine and Hugo W. Moser Research Institute at the Kennedy Krieger Institute, Baltimore, Maryland, United States of America; 3 Department of Medicine, Johns Hopkins University School of Medicine, Baltimore, Maryland, United States of America; 4 Department of Pathology, Johns Hopkins University School of Medicine, Baltimore, Maryland, United States of America; 5 Department of Cancer Biology, The Scripps Research Institute, Scripps Florida, Jupiter, Florida, United States of America; 6 Abramson Cancer Center, University of Pennsylvania, Philadelphia, Pennsylvania, United States of America; Roswell Park Cancer Institute, United States of America

## Abstract

The functions of key oncogenic transcription factors independent of context have not been fully delineated despite our richer understanding of the genetic alterations in human cancers. The MYC oncogene, which produces the Myc transcription factor, is frequently altered in human cancer and is a major regulatory hub for many cancers. In this regard, we sought to unravel the primordial signature of Myc function by using high-throughput genomic approaches to identify the cell-type independent core Myc target gene signature. Using a model of human B lymphoma cells bearing inducible *MYC*, we identified a stringent set of direct Myc target genes via chromatin immunoprecipitation (ChIP), global nuclear run-on assay, and changes in mRNA levels. We also identified direct Myc targets in human embryonic stem cells (ESCs). We further document that a Myc core signature (MCS) set of target genes is shared in mouse and human ESCs as well as in four other human cancer cell types. Remarkably, the expression of the MCS correlates with *MYC* expression in a cell-type independent manner across 8,129 microarray samples, which include 312 cell and tissue types. Furthermore, the expression of the MCS is elevated in vivo in Eμ-*Myc* transgenic murine lymphoma cells as compared with premalignant or normal B lymphocytes. Expression of the MCS in human B cell lymphomas, acute leukemia, lung cancers or Ewing sarcomas has the highest correlation with MYC expression. Annotation of this gene signature reveals Myc's primordial function in RNA processing, ribosome biogenesis and biomass accumulation as its key roles in cancer and stem cells.

## Introduction

Key oncogenic pathways are being revealed through sequencing of the human cancer genomes. While genetic alterations in specific human cancers continue to accumulate through deep sequencing, whether key oncogenic hubs, such as the MYC oncogene, behave in a general way or in a context dependent manner remains poorly understood. For that matter, whether alterations in oncogenic transcription factors result in cell-type independent effects has not been fully appreciated. In this regard, we focus on the MYC oncogene, which produces the Myc transcription factor involved in no less than 50% of human cancers, and seek to identify a putative cell-type independent target gene signature that might reveal Myc's primordial function in metazoans.

The c-*myc* (*MYC*) proto-oncogene was discovered over two decades ago as the cellular homolog of the retroviral v-*myc* gene that is sufficient to cause a variety of tumors in chickens [1], [2]. Deregulated expression of human *MYC* by multiple mechanisms contributes to human malignancies. Indeed, *MYC* is the most frequently amplified human oncogene and its activation by chromosomal translocation is a hallmark of Burkitt lymphoma [3]. Although *MYC* belongs to a family of related genes, including *MYCN* and *MYCL*, *MYC* is the most frequently activated member in human cancers. *MYCN* is well-known to be amplified in Stage IV neuroblastoma, and children with highly amplified *MYCN* suffer aggressive, poor prognosis disease [4].

In the mouse, homozygous deletion of *c-Myc* results in embryonic lethality, and conditional loss of *c-Myc* leads to tissue specific dysfunctional stem cell or committed progenitor compartments [5]. Mouse skin stem cells require *c-Myc* for differentiation into keratinocytes [6], and *c-Myc* is necessary for T lymphocyte progenitors to commit toward mature T cell compartments [7]. Hence, in tissue-specific stem cells *c-Myc* directs early rounds of cellular proliferation preceding terminal differentiation required for generating functional cell types. Ectopic *MYC* expression in normal cells, particularly lymphocytes, results in the activation of checkpoints, such as p53, Arf or BimL, which cause apoptosis and/or cell cycle arrest [8], [9]. Furthermore, enforced *Myc* expression in transgenic mice frequently results in hyperproliferation associated with apoptosis of the targeted tissue, and frank tumors arise following loss-of-function mutations in these checkpoints.

Intriguingly, Myc belongs to a cast of four transcription factors, including Sox2, Oct4 and Klf4, which together are sufficient to induce pluripotent stem cells (iPSCs) from somatic cells [10]. The iPSCs behave nearly identically to embryonic stem cells (ESCs), and removing Myc from this cocktail drastically reduces the efficiency of the induction of pluripotency [11]. Although *MYC* activates miRNAs that suppress mESC differentiation, the roles of *MYC* in establishing and maintaining pluripotency remain poorly understood [12], [13]. It is notable that ESC proliferation rates are faster than those of somatic cells, with mouse ESCs (mESCs) doubling as fast as every 5 hours and human ESCs (hESCs) do so every 16 hours [14], [15]. The cell cycles of both mESCs and hESCs have shortened G1 phases [15], in which ribosome biogenesis contributes significantly to the acquisition of a critical cell size for S-phase entry [16].

Myc associates with its partner, Max, forms a heterodimer, and regulates transcription by binding to canonical or related Myc consensus binding sites, with a preference for E-boxes having the sequence 5′-CACG/ATG-3′ [2], [17], [18], [19]. Upon binding, Myc-Max recruits cofactors that activate transcription largely through release of transcriptional pause [20]. Our previous mapping of Myc target sequences by chromatin immunoprecipitation and paired-end tag (ChIP-PET) sequencing revealed almost 3000 high quality binding sites, and predicted an estimated total of 6000 functional sites in the human model B cell genome [21]. Here we report, for the first time, Myc binding sites in hESCs. Due to unsaturated sequencing of our previous ChIP-PET library, we re-mapped Myc binding sites in human promoters in the P493-6 B cell. We have also measured genes that respond to *MYC* activation in P493-6 B cells by measuring both transcriptional rates using genome-wide nuclear run-on and steady-state RNA levels using microarray analyses of total RNA [22]. In addition, we have compared our MYC target genes with MYC targets in three cancer cell lines identified by ENCODE ChIP-seq data. Collectively, these analyses revealed a core set of direct Myc target genes that are shared in P493-6 B cells and in hESCs and mESCs, as well as in the human tumor HeLa, K562, and HepG2 cells. Importantly, analysis of MYC and this core target gene set in 8129 microarray samples from 312 different cell types showed a strong correlation between MYC expression and expression of its core target gene set in spite of the heterogeneity of samples.

Gene ontology analysis further revealed that the core Myc target genes are enriched with genes involved in ribosomal biogenesis, nucleolar function, and RNA processing. This Myc core signature is conserved from *Drosophila* to man and reveals a primordial function of *MYC* involving ribosomal biogenesis, consistent with both the nucleolar hypertrophy observed in ESCs and cancer [23], [24], [25] and with small cell and body sizes of adult *Drosophila diminutive* mutant flies having hypomorphic *dMyc* alleles that phenocopy loss of ribosomal protein function in *Drosophila* [26]. Although Myc's regulation of ribosomal biogenesis has been implicated in a few specific biological settings in previous studies [27], [28], [29], [30], our comprehensive unbiased analysis of data from many cell types in two species firmly establishes this role of Myc to be cell type and species independent. Moreover, for the first time, our analysis identifies this role of Myc in embryonic stem cells, multiple known MYC-driven cancers, and particularly in other previously un-suspected cancers. Our analysis of 8129 gene expression samples suggests that the 51 stringently defined cell type and species independent MYC core target genes can provide a signature for future screening of MYC pathway activities in diverse cell types and diseases.

## Results

### Identification of the Myc target gene signature and cell type-specific target genes

**Figure 1:**
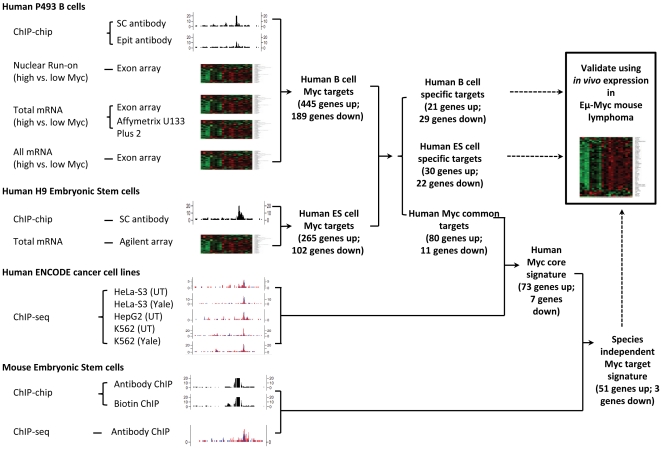
Schema of the strategy to identify the MYC core target gene signature.

To identify a species and cell type independent core set of Myc target genes we rigorously re-defined Myc target genes in the P493-6 human B cell model of Burkitt lymphoma containing an inducible *MYC* transgene [31], [32]. With the ability to regulate Myc expression level, we identified Myc targets by chromatin immunoprecipitation (ChIP) using a promoter array (ChIP-chip), global array-based nuclear run-on assay (ANRO) to measure changes in transcriptional rates [22], and multiple biological replicates for gene expression changes upon Myc induction. Genes that are bound by Myc, responded transcriptionally, and that had significant changes in total mRNA levels are considered direct Myc responsive target genes. With this target gene set in hand, we then identified Myc targets in the human embryonic stem cell line H9 by ChIP-chip and gene expression changes as discussed below. The overall strategy to derive a core set of Myc target genes independent of cell type and species that we designate the Myc Core Signature (MCS), and to identify cell-type specific Myc target genes is depicted in **Figure 1** (see **Table S1** for data sets analyzed, **Table S2** for complete expression and binding analysis, **Table S3** for the MCS and cell type specific target lists, and **Methods S1** for detailed data analysis procedures).

### Identification of direct MYC target genes in a human B cell model of Burkitt lymphoma

**Figure 2:**
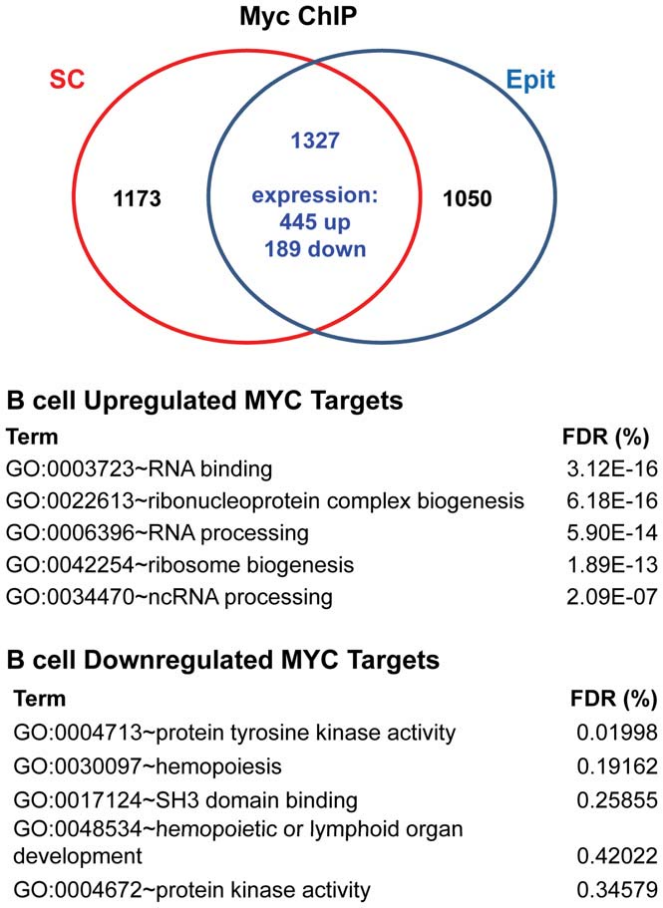
Direct Myc target genes in P493-6 B cells defined by ChIP-chip, array-based nuclear run-on, and gene expression changes. Venn diagram illustrating Myc bound target genes identified with Santa Cruz (SC) or Epitomics (Epit) anti-Myc antibodies. Bound genes whose expression changed are indicated for the overlap between SC and Epit. Enriched gene ontologies for these genes are shown.

We combined data derived from Myc ChIP with promoter tiled arrays, genome-wide array-based nuclear run-on to identify transcriptionally regulated genes, and changes in total mRNA levels to rigorously define direct Myc target genes and to exclude those whose expression might be altered at a post-transcription level. To determine the direct binding targets of Myc, ChIP was performed using two different anti-Myc antibodies in human P493-6 B cells, a model of Burkitt lymphoma that have an Epstein-Barr virus genome and a tetracycline (tet)-repressible human *MYC* transgene. Following removal of tet, Myc protein is highly induced and resting P493-6 cells are recruited into the active cell cycle [31]. Purified ChIP DNA from cells with high Myc levels was amplified and hybridized to Affymetrix human promoter 1.0R arrays. Signals from both Myc IP and IgG controls were normalized, and binding regions were detected and visualized using CisGenome [33]. In these B cells, the Santa Cruz (SC) anti-*N*-terminal Myc antibody revealed Myc binding to 2357 regions within 2500 genes with a false discovery rate (FDR) of 6.75%, while the Epitomics (Epit) monoclonal anti-*N*-terminal Myc antibody revealed 2303 bound regions within 2377 genes (FDR = 6.82%). Intersection of these two data sets revealed that Myc binds to the same regions in 1327 genes with two different anti-Myc antibodies (**Figure 2**).

We previously observed that Myc binding is often insufficient to trigger a change in gene expression, as only about 700 of the 3000 Myc bound genes identified by ChIP-PET had altered (induced or repressed) mRNA levels following Myc induction [21]. The same phenomenon was observed in Myc bound targets in mESCs, where genes that change in expression as a function of pluripotency have multiple bound transcription factors, such as Sox2, Oct4, Nanog, and Klf4 along with Myc [34]. As such, we coupled Myc target site identification by ChIP-chip on tiled promoter arrays with transcriptional changes using our global nuclear run-on studies of P493-6 B cells in the presence and absence of Myc induction. Furthermore, we compared this set of Myc-bound, transcriptionally regulated genes to the set of genes whose total RNA levels changed (as determined by both standard expression and exon microarrays) with Myc induction for overlaps. Combining these three methods identifies, for the first time, a stringent set of direct Myc responsive genes in human B cells (**Figure 1**). The overlap reveals a set of 634 genes, 445 of which are induced by MYC (**Figure 2** **and Table S3**). Gene ontology analysis using EASE [35] reveals that genes involved in RNA binding and processing as well as ribosome biogenesis are some of the functional classes that are over-represented in this set of Myc targets (**Figure 2**).

### Identification of a shared signature of MYC bound target genes in human B cells and embryonic stem cells

**Figure 3:**
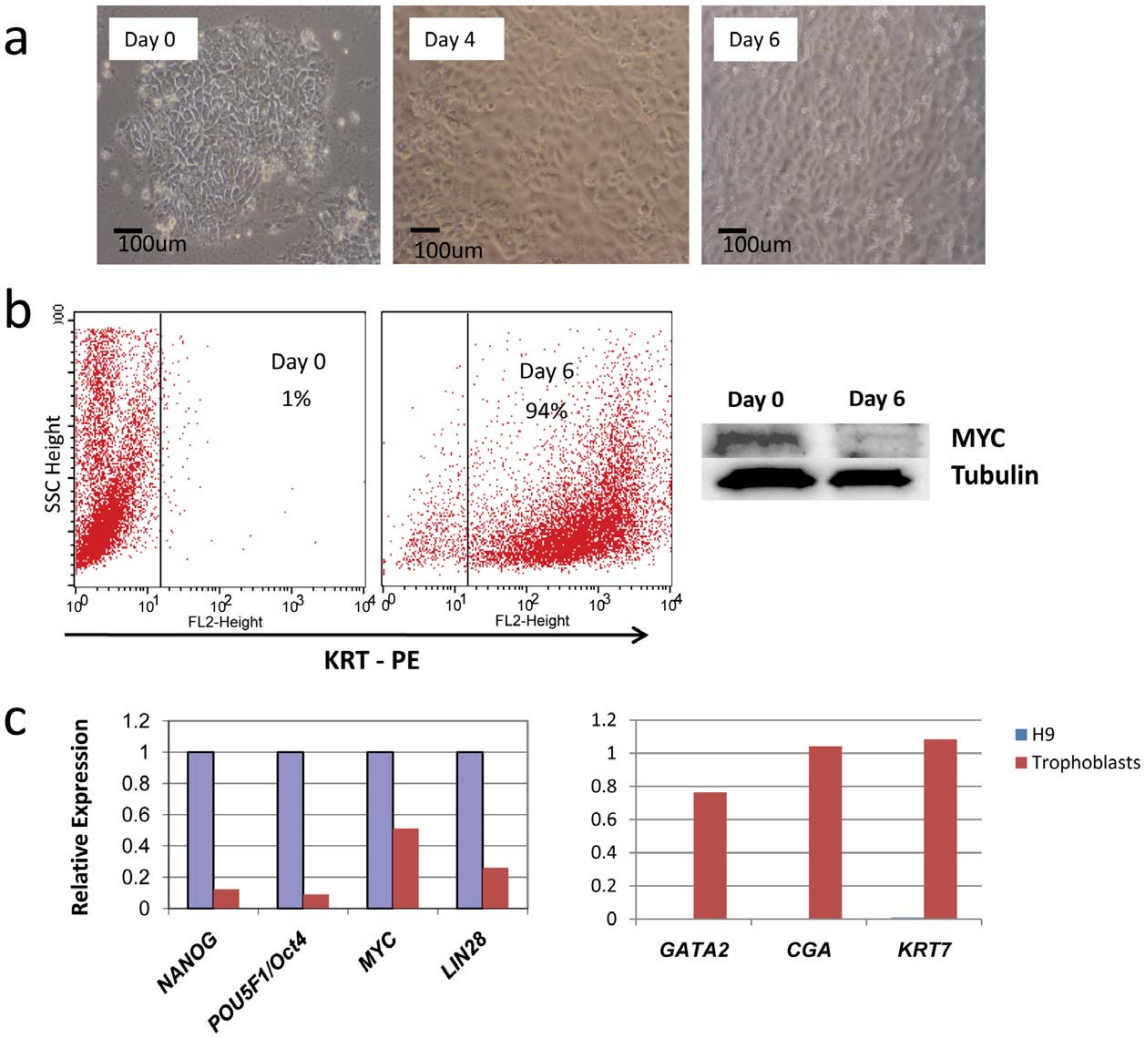
Trophoblastic differentiation of H9 hESCs is associated with reduced Myc. (**A**) Photomicrographs of H9 hESCs following exposure to BMP4 for the indicated times. Representative images are shown. (**B**) Flow cytometry of BMP4 treated cells stained for KRT-PE, a marker of trophoblastic differentiation. Immunoblot at right shows that Myc protein levels are drastically reduced in BMP4-treated H9 cells. Tubulin immunoblot shows equal loading of samples. (**C**) Expression of the indicated stem cell genes and those enriched in trophoblasts was determined by realtime PCR normalized to *B-ACTIN*.

MYC plays essential roles in stem cell pluripotency and self renewal, and in reprogramming adult cells to an induced pluripotent state [36]. However, the mechanisms by which Myc directs the induction of pluripotency are poorly understood. To define *MYC*'s functional role in ESCs, we determined direct Myc transcriptional targets in these cells. In hESCs, Myc levels are high and decrease with differentiation. Embryoid body formation promotes differentiation of stem cells into all three germ layers and is an attractive model to study the multipotentiality of hESC, but this is inherently limited by the cellular heterogeneity [37]. We therefore chose a simple, robust model that drives a more homogenous differentiation program, specifically using the bone morphogenetic protein BMP4 that induces differentiation of hESCs to trophoblasts, the cells that comprise the placenta [38]. After 6 days of exposure to BMP4, greater than 80% of cells differentiate and *MYC* mRNA and protein levels dropped several fold (**Figures 3B and 3C**), allowing us to study the Myc responsive transcriptome in a more uniform cell population. BMP4-treated H9 hESCs acquired a cobblestone appearance characteristic of differentiated trophoblasts (**Figure 3A**), and expressed high levels of *CGA*, *GATA2* and *KRT7* and reduced levels of pluripotency markers. Flow cytometric analyses revealed >90% of cells expressed high KRT7 levels compared to untreated H9 ESCs (**Figure 3B**).

To measure global gene expression changes associated with *MYC*, we isolated total RNA from H9 ESCs treated for 0 or 6 days with BMP4 and hybridized labeled cDNA to human gene expression arrays. We validated by qPCR the expression of the pluripotency markers *OCT4* (*POU5F1*) and *NANOG* as well as genes highly expressed in trophoblasts such as *GATA2*, *CGA* and *KRT7* that are virtually absent in undifferentiated H9 cells (**Figure 3C**). A subset of genes differentially expressed in undifferentiated hESCs versus trophoblasts should contain Myc target genes, where Myc-induced hESC target genes should be down-regulated following differentiation.

**Figure 4:**
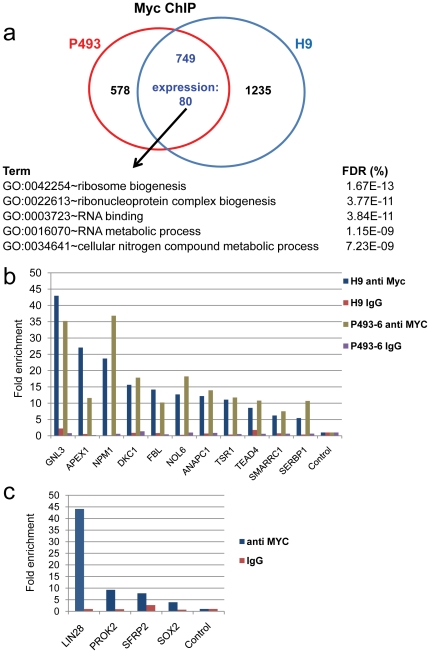
Myc target genes common to P493-6 B cells and H9 human embryonic stem cells. (**A**) Venn diagram of Myc bound genes in P493-6 and H9 hES cells with the overlap containing 80 upregulated genes. The ontology of these shared genes is shown. (**B**) RT-PCR validation of Myc-bound target genes in P493-6 and H9 cells with anti-Myc or control IgG signals shown. (**C**) Validation of Myc-bound targets in H9 hESCs by RT-PCR.

**Figure 5:**
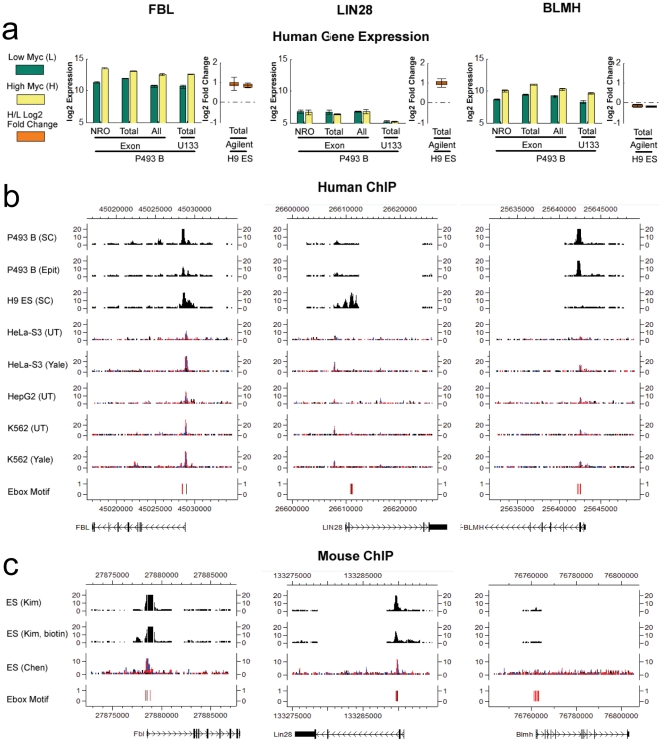
Examples of cell type independent, human ES cell and B cell specific Myc targets. (**A**) Gene expression levels (at log2 scale) of *FBL*, *LIN28* and *BLMH* in the absence and presence of Myc induction in P493-6 B cells (measured by Affymetrix Exon arrays and Affymetrix U133 Plus 2.0 arrays), and log2 gene expression fold changes between hESC and trophoblasts (measured by Agilent microarrays) are shown. Error bars correspond to standard deviations of replicate samples. (**B**) ChIP-chip binding signals in human P493-6 B cells and H9 ES cells, and ENCODE ChIP-seq binding signals in three human cancer cell lines. For ChIP-chip, TileMap moving average statistic [62] *m* was computed for each probe using normalized log2 probe intensities, and 2*^m^* was displayed as the intensity measure. For ChIP-seq, a 100 bp sliding window was used to scan the genome. Read count in each window was shown at a 25 bp step size. E-box motifs CACGTG (black) and CANNTG (red) were mapped to peak regions and are also shown. (**C**) ChIP-chip [34] and ChIP-seq [40] binding signals in mouse ES cells.

To identify potential Myc direct target genes in hESCs, we performed ChIP-chip in H9 hESCs using one of the anti-Myc antibodies (Santa Cruz) and identified 1787 binding regions within 1984 genes (FDR = 7.16%). The intersection of these Myc-bound hESC targets expressed in undifferentiated hESCs with the Myc direct targets in B cells reveals a common set of 80 up-regulated genes (**Figure 4A** **and Table S3**). We selected 11 genes from this set for qPCR validation and in all cases found significant binding to these promoters by Myc (**Figure 4B**). An example of the expression and Myc ChIP-chip data for the target gene fibrillarin (FBL) common to P493-6 B cells and H9 hESC are shown in **Figure 5**. Gene ontology analysis using EASE [35] reveals that the set of 80 core up-regulated human Myc targets contains genes involved with ribosome and ribonucleoprotein complex biogenesis, as well as RNA binding and metabolic processes (**Figure 4A**).

### Identification of a stringent set of cell type and species independent MYC core target genes

Next, we determined whether these 80 core Myc target genes are also bound by Myc in well-established human cancer cell lines. With the ENCODE Consortium data [39] which provide mapping of Myc binding sites by ChIP and direct sequencing (ChIP-seq) in three human cancer cell lines, HeLa (cervical cancer), K562 (chronic myelogenous leukemia) and HepG2 (liver cancer), we defined a common set of 73 genes among 5 human cell lines (**Table S3**). Notably, gene ontology analysis of this 73 gene set reveals ribosomal biogenesis and RNA processing as the two top cellular processes involved.

To identify a species-independent set of core MYC target genes, we assessed MYC binding to these genes using data from previous studies of Myc binding sites in mouse ESCs (**Table S1**). We re-analyzed raw ChIP-chip data from native Myc and in vivo biotinylated Myc proteins as well as ChIP-sequencing from two different studies and found that 51 of the 73 up-regulated genes are also bound in mESCs [34], [40]. Note that the Myc target gene *FBL* is also bound by Myc in mESC (**Figure 5C**). Thus, this core set of target genes that are bound by MYC in five different human cells lines and in mouse embryonic stem cells represents a cell type and species independent Myc Core Signature (MCS).

### Cell type restricted MYC targets

In addition to delineating the cell type-independent MCS, our analyses also provided an opportunity to delineate Myc target genes that are cell-type restricted to the human B P493 cells or hESCs, the two systems where we have expression data. Overall, 1523 genes are significantly down-regulated in trophoblasts while 1361 increase in expression as compared with undifferentiated hESCs. Of the genes more highly expressed in undifferentiated hESCs but that are not upregulated in Myc-expressing P493 cells, 30 are bound by Myc in H9 hESC but not in P493-6 B cells; thus, these represent direct MYC transcription targets that are specific to hESC. Two very interesting novel ES specific human Myc targets are *SOX2* and *LIN28* which are both important for maintaining pluripotency and reprogramming (**Figures 4** **and** **5**). For example, significant Myc binding to LIN28 is manifest in ESCs but not in other cells assessed in **Figure 5**. EASE analysis further reveals that genes involved with cell fate, cell-cell signaling, and signal transduction are statistically overrepresented in this hESC-specific MYC target gene set (FDR<22%).

To identify a core set of MYC target genes that are restricted to human B cells, we utilized P493-6 cells. We compared the transcriptional profile of proliferating cells with that of cells treated for 48 hr with tetracycline when these cells have very low MYC levels and are not cycling. Of the 522 expressed genes that are bound by MYC exclusively in this B cell model 9.6% of Myc targets were B cell specific, with 21 increasing with high MYC and 29 being repressed (**Table S3**). An example of a B cell selective Myc target is *BLMH* (**Figure 5**). Enriched in the list of induced B cell-specific Myc targets are genes involved in cellular organization, protein metabolism, mitochondria and generation of metabolites and energy. Among the direct B cell-specific targets suppressed by MYC are genes involved in lymphocyte activation, immune system development, leukocyte differentiation and hematopoiesis. Thus, MYC appears to maintain P493-6 B cells in an undifferentiated state by suppressing genes that direct lymphocyte maturation.

### Role of MCS in the human iPS cells, Eμ-*Myc* mouse and human lymphomas

Having identified a core set of Myc target genes shared between mouse and human pluripotent stem cells and a variety human cancer cells, the MCS, we sought to determine the behavior of these genes in human iPS cells. To determine if the MCS is also manifest in human iPSCs versus precursor fibroblasts, we examined the expression profiles of these targets using the publically available Amazonia website (http://amazonia.transcriptome.eu/ [41]) that annotates gene expression amongst different human cells and cell lines. Among the 51 core genes, 43 are significantly induced in iPSCs compared to the normal fibroblast counterparts, suggesting that these genes are involved in the pluripotent stem cell state (**Figure S1**).

**Figure 6:**
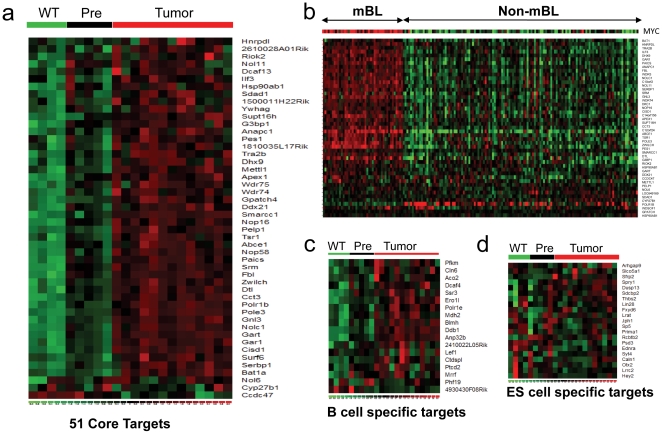
Myc core target gene signature increases with the neoplastic switch to frank lymphoma in vivo. (**A**) Heatmap showing expression levels of the 51 Myc core target genes in wild-type, pre-malignant Eμ-*Myc* (4–6 week old) littermates and of Eμ-*Myc* lymphoma. (**B**) Clustering of human molecular Burkitt's lymphoma (mBL) and non-mBL samples using the 51 core target genes. (**C**) and (**D**) Heatmaps showing expression levels of B cell restricted (**C**) or ES cell restricted (**D**) upregulated Myc target genes in the mouse B cell samples. Green – low expression; Red – high expression.

We then studied the behavior of the MCS *in vivo*, in a transgenic model of Myc-driven lymphoma. We used the Eμ-*Myc* mouse model from which one can directly compare expression profiles of wild type versus Myc-expressing B220+ pre-malignant lymphocytes and also query differences in gene expression that ensue following the neoplastic switch to frank lymphoma [42]. Cluster analysis of expression of the 51 gene (of which we found murine probe sets for 48 genes) signature amongst wild type, premalignant Myc and malignant Myc B lymphocytes reveals a distinct increased expression of the MCS that correlates with the transformed malignant phenotype (**Figure 6A**). Further analysis showed that while 24.9% (6212 out of 24944) of genes on the microarray had increasing expression levels from wild type to premalignant Myc to malignant Myc B lymphocytes, 91.7% of the MCS (44 of the 48 genes) followed this pattern (**Figure 6A**, **Methods S1**), representing a much higher percentage (>3-fold enrichment). The overall correlation indicates that the MCS contributes to the malignant state. As predicted from analyses of P493-6 cells and hESCs, GSEA analysis established that the “Ribosome Pathway” gene set (http://www.broadinstitute.org/gsea/msigdb/cards/HSA03010_RIBOSOME.html) is also significantly enriched in premalignant Eμ-*Myc* B220^+^ B cells compared to their wild type counterparts (FDR = 0.26%, **Figure S2**). Finally, when 173 human mature aggressive B lymphoma samples were used for the clustering of the 51 gene MCS [43], molecular Burkitt's lymphoma (mBL) cases have distinctly higher MYC and MCS expression than the non-mBLcases (**Figure 6B**). With unsupervised clustering (**Figure S3**), all 44 mBL cases grouped together along with 10 out of 129 non-mBL cases, which have higher MCS expression. This observation suggests that some non-mBL cases in this series have higher MYC and MCS expression.

We further sought to determine the significance of the cell type restricted Myc target genes found in hESCs versus those found specifically in human P493-6 B lymphocytes. When P493-6 B cell-specific Myc target genes were used for analysis of mouse gene expression from wild type, premalignant Myc or malignant Myc B cells, the vast majority (15 out of 18, or 83.3%) of the B cell specific Myc-induced genes increased in this transgenic lymphoma model (**Figure 6C**) while 73.9% (17 out of 23) repressed genes were decreased (**Figure S4**). On the other hand, the hESC-specific Myc target genes did not provide the same pattern amongst the B lymphocytes from wild type or Myc transgenic mice, with only 3 (14.3%) out of the 21 positive targets displaying the increasing pattern (**Figure 6D**) and 5 (31.3%) out of the 16 repressed targets displaying the decreasing pattern (**Figure S4**).

### Correlation of the MCS with MYC expression in 8000 microarray samples

**Figure 7:**
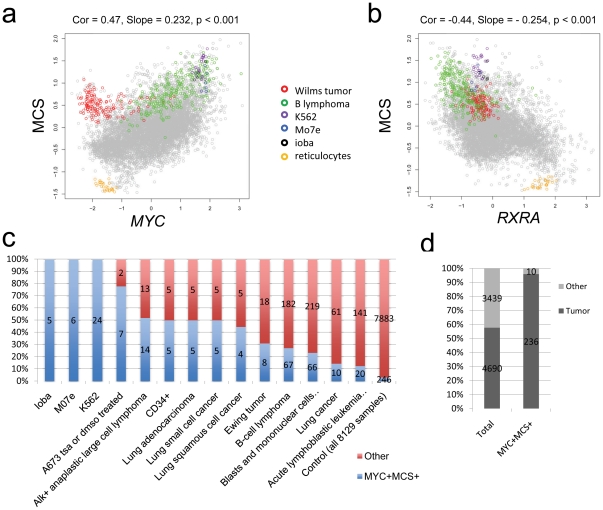
Correlation of *MYC* and Myc core target gene signature (MCS) expression among 8129 samples. (**A**) Correlation plot of MCS versus *MYC* expression across 8129 samples on the Affymetrix human U133A platform. Correlation coefficient = 0.47. p-value was determined based on correlation between *MYC* and randomly chosen genes (see **Methods S1**). Samples from a few cell or tissue types exhibiting patterns of interest are highlighted in color. (**B**) Correlation plot of MCS versus *RXRA* expression across 8129 samples on the Affymetrix U133A platform. (**C**) Total percentage and samples counts of each significantly enriched (FDR<5%) high *MYC* high MCS cell or tissue type in MYC+ MCS+ samples compared to in all samples. (**D**) Comparison of total percentage of tumor samples in all of the data (57.7%) to the total percentage of MYC+ MCS+ samples that are tumors (95.9%).

To further determine the cell-type independence of the MCS, we assessed its expression in a collection of 8129 Affymetrix human U133A microarray samples stored in Gene Expression Omnibus [44], representing about 312 different cell and tissue types. We hypothesized that if the core targets are cell type independent, one should be able to observe a clear correlation between the gene expression level of the MCS and *MYC* expression across this large collection of microarray samples with different cell types. Intriguingly, we observe a strong and highly statistically significant correlation of MCS expression with *MYC* (**Figure 7A**, correlation coefficient = 0.47, p-value<0.001). A small cluster of samples (red circles) enriched in Wilms tumor have low MYC but high MCS for reasons unknown to us (possible explanations include other transcriptional regulators, such as N-Myc, that may activate the same set of genes, lab/batch effects in the microarray experiments, or others). However, other than these samples, the correlation between MYC and MCS are strikingly strong given the heterogeneity of the samples involved in the analysis. Interestingly, when we evaluated the expression of the MCS with that of RXRα, a transcription factor that drives differentiation [45], we observe an inverse correlation (**Figure 7B**).

The use of the MCS to scan the large collection of microarray samples representing both normal and cancer cells and tissues provides a unique opportunity to survey the various cellular contexts, in which Myc regulation of the MCS is active. In addition to B cell lymphoma, we identified other cell types having elevated expression of both MYC and MCS and found that 95.9% (236/246) of these are various types of tumors, while only 57.7% (4690/8129) of the total samples are tumors (**Figure 7C, D**). All cell types shown in **Figure 7C** have statistically significant fractions of samples (FDR<5%) that display both high MYC and MCS expression when compared to randomly selected samples. Among the identified tumors with high *MYC* and MCS expression, several including acute lymphoblastic leukemia have been associated with high *MYC* expression. For some tumors (e.g. Ewing Sarcoma), the functional role of MYC pathway has not yet been established.

The correlation between MYC and MCS in this very large collection of gene expression profiles from diverse cells and tissues underscores the importance of this cell type independent core set of Myc target genes. Furthermore, this analysis provides the first comprehensive microarray data-driven survey of cellular contexts in which MYC might play an important role in regulating genes involved in ribosome biogenesis.

## Discussion

### Regulation of the MCS genes involved in nucleolar function and ribosomal biogenesis reveals Myc's primordial function

Our comprehensive analysis of genes directly regulated by Myc reveals a cell type and species independent signature with functions in ribosome biogenesis and nucleolar proteins involved in RNA processing. This finding unifies many previous observations linking Myc to ribosome biogenesis in diverse mammalian cell types and in *Drosophila* and underscores the importance of nucleolar hypertrophy as a hallmark of cancer, which was originally described in 1896 [23], [27], [28], [29], [30], [46], [47]. This hallmark remains a distinctive feature of cancer cells, particularly for prognosis. In a separate study, we found that MYC regulates a number of genes and gene sets related to ribosome biogenesis and nucleolar function in human prostate cancer cells, and that fibrillarin, which is one of 51 MCS genes, is necessary for prostate cancer cell proliferation [48]. Fibrillarin levels correlate with Myc levels in both the human prostate cancer precursor lesions, high grade prostatic intraepithelial neoplasia (PIN), and in various stages of human prostate cancer. This correlation provides a molecular underpinning for increased nucleolar hypertrophy that occurs during all phases of prostatic neoplasia [49].

The functional role of ribosomal biogenesis in Myc-mediated tumorigenesis has been established through the effects of *Rpl24* heterozygosity on Eμ-*Myc* lymphomagenesis [25], [50], where Eμ-Myc;*Rpl24* ^+/−^ mice have reduced spleen sizes, delayed lymphoma development and prolonged survival compared to their Eμ-Myc;*Rpl24* ^+/+^ littermates. Thus, normal ribosomal biogenesis is essential for efficient tumorigenesis.

The overall function of the MCS suggests a primordial function for Myc during evolution. *Drosophila* dMyc hypomorphs have phenotypes of loss of ribosomal function, resulting in small cell and body size [26]. Intriguingly, Myc was recently found to be expressed in the interstitial stem cells of Hydra, an ancient marine metazoan that has existed for over 600 million years [51]. Because interstitial stem cells participate in regeneration of the Hydra polyp and Myc is highly localized to the Hydra stem cell compartment, Myc is likely to be central to tissue regeneration in this organism. We expect that the primordial function of Myc in ribosome biogenesis can also be found in Hydra through a homologous Myc target gene signature.

### Target gene context and cell type specificity

An early study, focused on the transcriptional circuitry of Oct4, Sox2 and Nanog in hESCs, found that these three transcription factors (TFs) co-occupy a substantial portion of their target genes [52]. The data also indicated that these TFs collaborate to form regulatory circuits consisting of autoregulatory and feed-forward loops. More recently, transcriptional regulation has been assessed in mouse ES cells by examining the promoters bound by nine TFs [34] and the genome-wide binding map of 13 sequence-specific TFs and two transcriptional regulators using ChIP-seq [40]. Interestingly, these studies revealed that target gene promoters bound by only a few TFs are largely inactive or repressed while those bound by four or more tend to be active in the pluripotent state, and to be repressed upon differentiation. Even though both of these extensive studies included Myc, they were conducted in mESCs. Here we provide insight into *MYC*'s role in human stem cells, where we have defined a stem cell specific set of Myc targets, which include *LIN28* and are found in both murine and human ESCs and in iPSCs, but not in human P493-6 B lymphocytes.

Recently, the *LIN28* family member *LIN28B*, whose expression in highly elevated in a number of human tumors, was shown to be a direct MYC target in P493-6 B cells and mouse lymphoma [53]. Lin28B inhibits the maturation of members of the *Let7* family of miRNAs that are predicted to repress MYC, completing an intricate autoregulatory loop important in both stem biology and tumorigenesis. It is intriguing to note that the three factors, Sox2, Oct4, and Klf4, can be complemented by either *MYC* or *LIN28* to induce more efficiently pluripotency in fibroblasts [54], [55]. As such, the role of Myc and LIN28 in the regulation of miRNAs, which are not determined in the current study, is also expected to play critical roles in the pluripotent stem cell state.

Our study also defined Myc target genes that are bound and regulated only in B cells and not in ESCs. The expression of B cell-specific Myc targets correlated well with in vivo expression of these genes in Eμ-*Myc* malignant lymphocytes as compared to the normal or pre-malignant counterparts. These observations indicate that our identification of B-cell specific genes in the human P493-6 model is relevant to Myc-driven lymphomagenesis, whereas ESC-specific genes do not appear to be relevant to *in vivo* lymphoma.

Most importantly, our comprehensive analysis of genome-wide ChIP-chip, ChIP-seq and gene expression data from multiple cell types allowed us to define a stringent set of cell type and species independent MYC core target genes. The expression of the MCS which is shared amongst six murine or human cell systems increased from normal to premalignant to neoplastic B cells. The fact that the expression of the 51 core set of Myc targets also correlates with *MYC* expression amongst 8129 gene expression profiles from diverse normal and diseased human cells and tissues underscores the cell type independent nature of this set. Among the tumors with high *MYC* and MCS expression, several including acute lymphoblastic leukemia have been associated with high *MYC* expression, whereas this association was not previously known for tumors like Ewing Sarcoma. Thus, this analysis opens up new biological avenues to investigate. The signature of this core 51 gene set is dominated by genes involved in ribosome biogenesis and RNA processing, revealing that Myc induces biomass accumulation in a cell type-independent fashion. In this regard, deregulated expression of MYC in cancers could cause constitutive biomass accumulation, rendering cancer cells addicted to nutrients such as glucose or glutamine similar to yeast mutants that were genetically engineered to have constitutive ribosome biogenesis [56], [57], [58].

## Materials and Methods

Additional experimental procedures are documented as Methods S1.

### P493-6 cell culture

P493-6 cells, originally established in [31], were cultured in RPMI with 10% fetal bovine serum and penicillin and streptomycin and treated with tetracycline (0.1 µg/ml) to silence exogenous *MYC* transgene expression as previously described [21], [32].

### Human ES cell culture and trophoblast cell differentiation

The H9 hES cells were purchased from the WiCell Research Institute and were initially cultured on PMEFs as instructed by the provider. H9 cells used in this experiment were at passage 30 and were karyotypically normal at the beginning of the experiment.

Differentiation of hESC in the presence of BMP4 was performed as previously described [38].

### Flow cytometric analysis

Trophoblast cells were dissociated with trypsin, fixed with 2% paraformaldehyde (fresh prepared), permeabilized with 0.1%triton X 100, blocked with 4% *γ* globulins, and stained with monoclonal antibody Cytokeratin 7 (Dako) as primary antibody and PE- anti mouse IgG1 (BD) as secondary antibody. Flow cytometric analysis was performed on a FACSCalibur instrument using the CellQuest software (BD Bioscience, San Jose, CA). At least 10,000 events were acquired for each sample.

### Chromatin Immunoprecipitation

Chromatin immunoprecipitation (ChIP) was performed on untreated P493-6 cells as previously described [21]. For H9 hESCs, the cells were treated with formaldehyde (1% final concentration) for 10 min on the plate. Chromatin from 10^7^ cells was incubated with 1 ug antibody. Antibody-chromatin complexes were pulled down with Staph A cells, washed, and formaldehyde crosslinks were reversed as previously described [21]. Antibodies used for ChIP were anti-MYC (Santa Cruz cat# sc-764, Epitomics rabbit monoclonal cat# 1472-1), IgG control (normal rabbit IgG, Santa Cruz cat# sc-2027). For ChIP-chip, 10% of the IP product from the MYC and IgG control pulldowns was used for amplification following Affymetrix ChIP-chip protocol. Amplified ChIP products were fragmented, labeled and hybridized to Affymetrix 1.0 Human Promoter arrays.

### RNA Extraction and Gene Expression Analysis

RNA was extracted from 1–5 million P493-6 (untreated and tet treated) and H9 (untreated and BMP4 treated) cells using a Qiagen RNeasy kit (cat# 74104). RNA samples were submitted to the Johns Hopkins Microarray Core Facility to be hybridized to Agilent Whole Human Genome 44K microarrays (H9 cells) or Affymetrix Human Genome U133 Plus 2.0 or Human Exon 1.0 ST Arrays (P493-6 cells). One ug RNA was converted to cDNA using the TaqMan Reverse Transcription Kit (Applied Biosystems, cat# N808-0234). cDNA was amplified using 250 nM gene specific primers and Power SYBR Green (Applied Biosystems, cat# 4367659). *MYC* and *B-ACTIN* was amplified using Taqman gene expression assays. Quantitation was performed using a relative standard curve. Expression level for each gene was normalized to *B-ACTIN*. PCR primer sequences are provided in **Table S4**.

### Data Analysis

Detailed data analysis methods and methods for derivation of the different sets of Myc targets are provided in Methods S1.

#### ChIP-chip and ChIP-seq Data Analysis

All ChIP-chip data (including human P493 B cell and H9 ES cell, and mouse ES cell data) were analyzed using CisGenome [33]. Details are provided in Methods S1.

#### Gene Expression Data Analysis

Affymetrix Exon array data (including nuclear run-on, total RNA and all RNA in human P493 B cells) were processed using the GeneBASE software [59]. RMA was used for processing of other microarray data. Details are provided in Methods S1.

#### Myc Targets in Human P493-6 B Cells and Human H9 ES Cells

Details for deriving common and cell type restricted Myc targets are provided in Methods S1.

#### Analysis of 8129 GEO Microarray Samples

8129 Affymetrix Human U133A microarray samples were collected from GEO [60]. The samples were normalized and processed using fRMA [61] to compute gene expression levels. Details are provided in Methods S1.

### Data Availability

All new microarray and ChIP-chip data generated by this study are submitted to Gene Expression Omnibus (GEO). GEO accession numbers: GSE32220, GSE32239.

## Supporting Information

Figure S1 **Expression of 43 Myc core signature genes in fibroblasts, human induced pluripotent stem cells (iPSC) or human embryonic cells (ESC).** The data were obtained from http://amazonia.transcriptome.eu/. Each vertical colored bar represents a single sample and the height corresponds to expression level of the indicated Affymetrix probe set.
(PDF)

Figure S2 **GSEA summary statistics of Ribosome pathway gene set demonstrate its significant enrichment (FDR = 0.26%) in premalignant Eμ-Myc B220+ samples as compared to wild type.** Profile of the Running Enrichment Score (ES) and position of gene set members on the rank ordered list show positive correlation with premalignant Eμ-Myc B220+ data.
(PDF)

Figure S3 **Unsupervised clustering of lymphoma samples with the 51 gene Myc core signature.** Note the clustering of all 44 molecular Burkitt lymphoma (mBL) samples together with 10 non-mBL samples. The remaining non-mBL samples cluster in a separate branch.
(PDF)

Figure S4 **Clustering of downregulated B-cell restricted or embryonic stem (ES) cell restricted Myc target genes among mouse samples from wild-type (WT), premalignant (Pre) or frankly malignant (Tumor) B220+ lymphocytes.**
(PDF)

Table S1 **ChIP-chip, ChIP-seq and gene expression data used in the analysis.**
(DOC)

Table S2 **Detailed ChIP-chip, ChIP-seq and gene expression data analysis results.**
(XLS)

Table S3 **Myc target gene lists.**
(XLS)

Table S4 **PCR primer sequences used in the study.**
(XLS)

Methods S1 **Supplemental Methods.**
(DOC)
