# Supplementary material for: Cell-Type Independent MYC Target Genes Reveal a Primordial Signature Involved in Biomass Accumulation
Source: PLoS One. 2011 Oct 19;6(10):e26057. doi: 10.1371/journal.pone.0026057 (PMC3198433; doi:10.1371/journal.pone.0026057)
Supplement: Figure S1 — Expression of 43 Myc core signature genes in fibroblasts, human induced pluripotent stem cells (iPSC) or human embryonic cells (ESC). The data were obtained from http://amazonia.transcriptome.eu/. Each vertical colored bar represents a single sample and the height corresponds to expression level of the indicated Affymetrix probe set. (PDF) [file pone.0026057.s001.pdf]

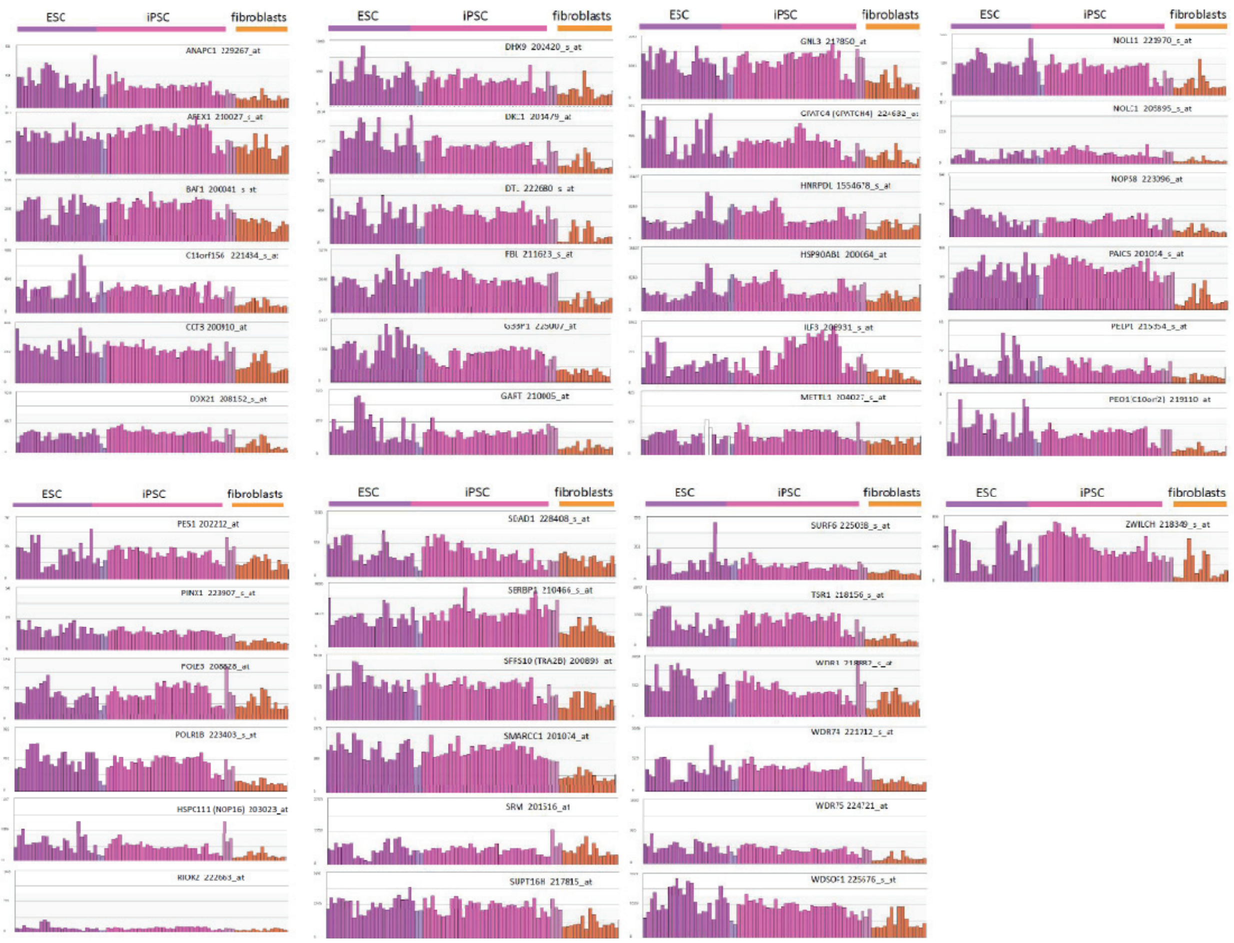

Figure S1. Expression of 43 Myc core signature genes in fibroblasts, human induced pluripotent stem cells (iPSC) or human embryonic cells (ESC). The data were obtained from http://amazonia.transcriptome.eu/. Each vertical colored bar represents a single sample and the height corresponds to expression level of the indicated Affymetrix probe set.
