# Supplementary material for: Cell-Type Independent MYC Target Genes Reveal a Primordial Signature Involved in Biomass Accumulation
Source: PLoS One. 2011 Oct 19;6(10):e26057. doi: 10.1371/journal.pone.0026057 (PMC3198433; doi:10.1371/journal.pone.0026057)
Supplement: Figure S2 — GSEA summary statistics of Ribosome pathway gene set demonstrate its significant enrichment (FDR = 0.26%) in premalignant Eμ-Myc B220+ samples as compared to wild type. Profile of the Running Enrichment Score (ES) and position of gene set members on the rank ordered list show positive correlation with premalignant Eμ-Myc B220+ data. (PDF) [file pone.0026057.s002.pdf]

Table: GSEA Results Summary

Dataset	preEuMyc-WT-RMA_collapsed_to_symbols.preEuMyc-WT-lables.cls#preEuMyc_versus_WT
Phenotype	preEuMyc-WT-lables.cls#preEuMyc_versus_WT
Upregulated in class	preEuMyc
GeneSet	HSA03010_RIBOSOME
Enrichment Score (ES)	0.5461467
Normalized Enrichment Score (NES)	1.8738376
Nominal p-value	0.002247191
FDR q-value	0.0026476353
FWER p-Value	0.041

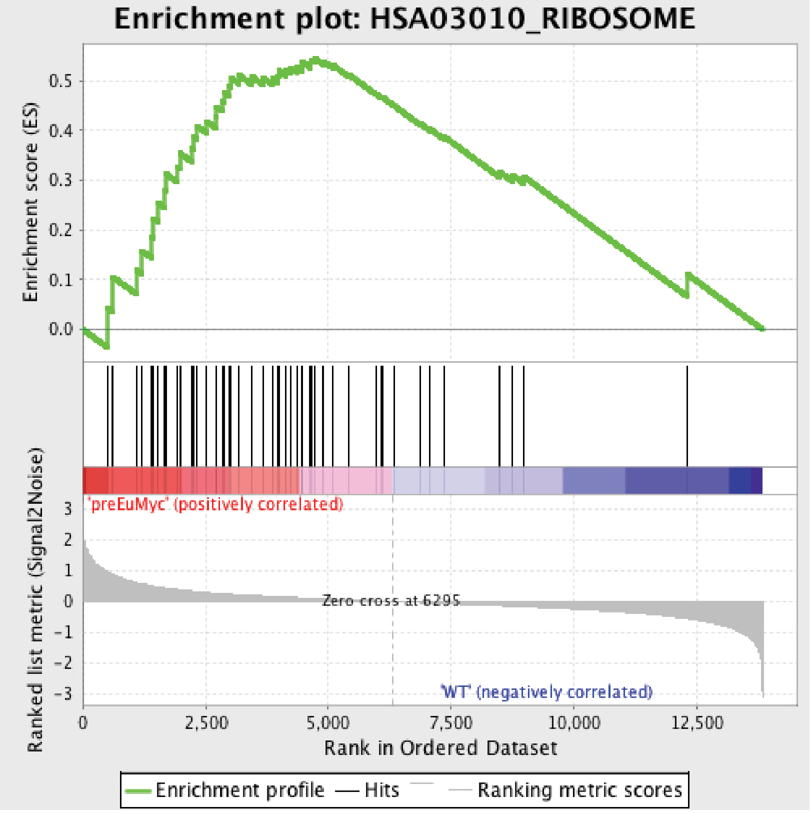

Figure S2. GSEA summary statistics of Ribosome pathway gene set demonstrate its significant enrichment (FDR = 0.26%) in premalignant Eμ-Myc B220+ samples as compared to wild type. Profile of the Running Enrichment Score (ES) and position of gene set members on the rank ordered list show positive correlation with premalignant Eμ-Myc B220+ data.
