# Supplementary material for: Cell-Type Independent MYC Target Genes Reveal a Primordial Signature Involved in Biomass Accumulation
Source: PLoS One. 2011 Oct 19;6(10):e26057. doi: 10.1371/journal.pone.0026057 (PMC3198433; doi:10.1371/journal.pone.0026057)
Supplement: Figure S3 — Unsupervised clustering of lymphoma samples with the 51 gene Myc core signature. Note the clustering of all 44 molecular Burkitt lymphoma (mBL) samples together with 10 non-mBL samples. The remaining non-mBL samples cluster in a separate branch. (PDF) [file pone.0026057.s003.pdf]

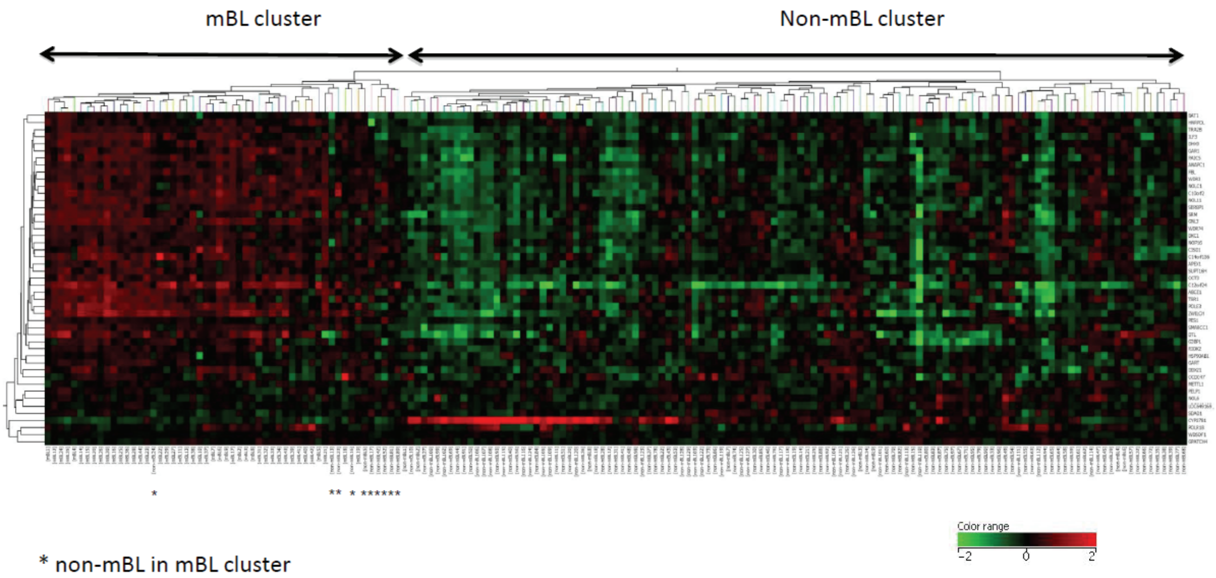

Figure S3. Unsupervised clustering of lymphoma samples with the 51 gene Myc core signature. Note the clustering of all 44 molecular Burkitt lymphoma (mBL) samples together with 10 non-mBL samples. The remaining non-mBL samples cluster in a separate branch.
