# Supplementary material for: Cell-Type Independent MYC Target Genes Reveal a Primordial Signature Involved in Biomass Accumulation
Source: PLoS One. 2011 Oct 19;6(10):e26057. doi: 10.1371/journal.pone.0026057 (PMC3198433; doi:10.1371/journal.pone.0026057)
Supplement: Figure S4 — Clustering of downregulated B-cell restricted or embryonic stem (ES) cell restricted Myc target genes among mouse samples from wild-type (WT), premalignant (Pre) or frankly malignant (Tumor) B220+ lymphocytes. (PDF) [file pone.0026057.s004.pdf]

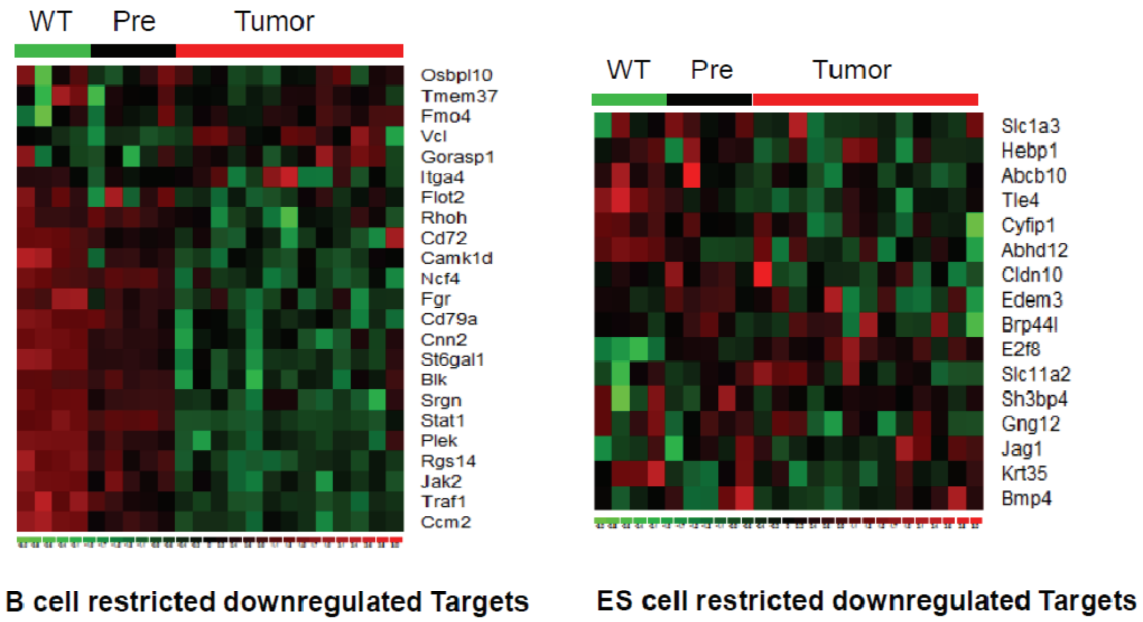

Figure S4. Clustering of downregulated B-cell restricted or embryonic stem (ES) cell restricted Myc target genes among mouse samples from wild-type (WT), premalignant (Pre) or frankly malignant (Tumor) B220+ lymphocytes.
