# Supplementary material for: Cell-Type Independent MYC Target Genes Reveal a Primordial Signature Involved in Biomass Accumulation
Source: PLoS One. 2011 Oct 19;6(10):e26057. doi: 10.1371/journal.pone.0026057 (PMC3198433; doi:10.1371/journal.pone.0026057)
Supplement: Table S1 — ChIP-chip, ChIP-seq and gene expression data used in the analysis. (DOC) [file pone.0026057.s005.doc]

**Supplemental Table S1. ChIP-chip, ChIP-seq and Gene Expression Data** Used in the Analysis

| **Data** | **Species** | **Type** | **Platform** | **Samples** | **Sources** |
| --- | --- | --- | --- | --- | --- |
| P493 B cells | Human | ChIP-chip (Santa Cruz antibody) | Affymetrix Human Promoter 1.0R Arrays | 6 (3 IP vs. 3 IgG control) | This study  (GEO:GSE32220) |
| P493 B cells | Human | ChIP-chip (Epit antibody) | Affymetrix Human Promoter 1.0R Arrays | 4 (2 IP vs. 2 IgG control) | This study  (GEO:GSE32220) |
| H9 hES cells | Human | ChIP-chip (Santa Cruz antibody) | Affymetrix Human Promoter 1.0R Arrays | 6 (3 IP vs. 3 IgG control) | This study  (GEO:GSE32220) |
| HeLa-S3 | Human | ChIP-seq (UT) | Illumina Genome Analyzer | 3 (2 IP vs. 1 Input) | ENCODE |
| HeLa-S3 | Human | ChIP-seq (Yale) | Illumina Genome Analyzer | 3 (2 IP vs. 1 Input) | ENCODE |
| HepG2 | Human | ChIP-seq (UT) | Illumina Genome Analyzer | 4 (3 IP vs. 1 Input) | ENCODE |
| K562 | Human | ChIP-seq (UT) | Illumina Genome Analyzer | 4 (3 IP vs. 1 Input) | ENCODE |
| K562 | Human | ChIP-seq (Yale) | Illumina Genome Analyzer | 4 (2 IP vs. 2 Input) | ENCODE |
| mES cells | Mouse | ChIP-chip (antibody ChIP) | Affymetrix Mouse Promoter 1.0R Arrays | 6 (3 IP vs. 3 Input) | Kim et al. (GEO:GSE11329) |
| mES cells | Mouse | ChIP-chip (biotin-mediated ChIP) | Affymetrix Mouse Promoter 1.0R Arrays | 7 (3 IP vs. 4 BirA control) | Kim et al. (GEO:GSE11329) |
| mES (E14) cells | Mouse | ChIP-seq | Illumina Genome Analyzer | 2 (1 IP vs. 1 GFP control) | Chen et al. (GEO:GSE11431) |

**Supplementary Table S1. (Cont.)**

| **Data** | **Species** | **Type** | **Platform** | **Samples** | **Sources** |
| --- | --- | --- | --- | --- | --- |
| P493 B cells | Human | Nuclear Run-on (NRO) | Affymetrix Human Exon 1.0 ST Arrays | 8 (4 low Myc [tet-treated] vs. 4 high Myc [untreated]) | Fan et al.  (GEO:GSE17239) |
| P493 B cells | Human | Total RNA | Affymetrix Human Exon 1.0 ST Arrays | 8 (4 low Myc [tet-treated] vs. 4 high Myc [untreated]) | Fan et al.  (GEO:GSE17239) |
| P493 B cells | Human | All RNA (NRO and Total) | Affymetrix Human Exon 1.0 ST Arrays | 6 (3 low Myc [tet-treated] vs. 3 high Myc [untreated]) | This study  (GEO:GSE32220) |
| P493 B cells | Human | Total RNA | Affymetrix Human Genome U133 Plus 2.0 Arrays | 12 (5 low Myc [tet-treated] vs. 7 high Myc [untreated]) | Yustein et al.  (GEO:GSE19703) |
| H9 hES cells | Human | Total RNA | Agilent  Whole Human Genome 44K Arrays | 6 (3 hESCs vs. 3 trophoblasts) | This study  (GEO:GSE32220) |
| B lymphoma | Mouse | Total RNA | Affymetrix Mouse Expression 430A Arrays | 22 (4 WT + 5 premalignant tumors + 13 malignant tumors) | Nilsson et al. (GEO:GSE32239) |
| B lymphoma | Human | Total RNA | Affymetrix  Human  Genome U133A  Arrays | 173 diffuse large B-cell lymphoma and Burkitt lymphoma | Hummel et al.  (GEO:GSE4475) |
