# Supplementary material for: Cell-Type Independent MYC Target Genes Reveal a Primordial Signature Involved in Biomass Accumulation
Source: PLoS One. 2011 Oct 19;6(10):e26057. doi: 10.1371/journal.pone.0026057 (PMC3198433; doi:10.1371/journal.pone.0026057)
Supplement: Methods S1 — Supplemental Methods. (DOC) [file pone.0026057.s009.doc]

**SUPPLEMENTAL INFORMATION**

**Cell-type independent MYC target genes reveal a primordial signature involved in biomass accumulation**

**Hongkai Ji, George Wu, Xiangcan Zhan, Alexandra Nolan, Cheryl Koh, Angelo De Marzo, Hoang Mai Doan, Jinshui Fan, Christopher Cheadle, Mohammad Fallahi,**

**John L. Cleveland, Chi V. Dang, and Karen I. Zeller**

**Supplemental METHODS**

**Quantitative Realtime PCR**

Realtime PCR was performed as described [1]. ChIP products were amplified using 250nM primers and Power SYBR Green (Applied Biosystems, cat# 4367659). Quantitation was performed using a relative standard curve and fold enrichment was calculated by dividing signal obtained from promoter binding regions by signals from a negative control region. PCR primer sequences are provided in **Supplemental Table S4.**

**ChIP-chip and ChIP-seq Data Analysis**

All ChIP-chip data (including human P493-6 B cell and H9 ES cell, and mouse ES cell data) were analyzed using CisGenome [2]. After quantile normalization [3], the TileMapv2 Moving Average (MA) algorithm [4] implemented in CisGenome was used to detect peaks using MA ≥ 3.0 as the cutoff. Myc binding regions in the ENCODE ChIP-seq data were downloaded from the ENCODE Data Coordination Center at UCSC (<http://genome.ucsc.edu/ENCODE/>) (ENCODE Project Consortium, 2007). For the present study, only those data that were not restricted for publication were used. ChIP-seq Myc binding regions in mouse ES cells were obtained from Chen et al. [5]. Myc binding regions were associated with nearby promoters based on the RefSeq transcript annotation. If a transcript has ≥1 Myc binding site within 10 kb upstream and 5 kb downstream of its transcription start site (TSS), the transcript is annotated as a Myc bound gene. All analyses were done using the human genome assembly hg18 and mouse genome assembly mm8.

**Gene Expression Microarray and Exon Array Data Analysis**

Affymetrix Exon array data (including nuclear run-on, total RNA and all RNA in human P493-6 B cells) were processed using the GeneBASE software [6]. GeneBASE performs background correction and normalization based on probe sequences. It then combines background corrected and normalized probe intensities within each gene to compute the gene’s expression level. Affymetrix microarray data (including total RNA in P493-6 B cells measured by Human U133 Plus 2.0 arrays, and total RNA in wild type and Eμ-*Myc* premalignant B220+ lymphocytes and in malignant Eμ-*Myc* lymphoma measured by Mouse 430A arrays) were processed using the RMA [7] algorithm to compute gene expression levels. Agilent data (total RNA in human H9 ES cells) were quantile normalized. The normalized probe intensities were then used as the gene expression levels. To determine differentially expressed genes, gene expression levels were log2 transformed and then analyzed using limma [8]. For Affymetrix Human Exon array and Human U133 Plus 2.0 array data, differential expression was called at the FDR = 5% cutoff. The Agilent gene expression data had a slightly higher noise level, and the differential expression was called using the FDR = 25% cutoff. Clustering analysis was performed using dChip [9,10].

**Myc Targets in Human P493-6 B Cells**

The promoter ChIP-chip analysis identified 2357 (FDR=6.75%) Myc binding regions in human P493-6 B cells using the Santa Cruz (SC) anti-*N*-terminal Myc antibody, representing 2500 genes. Using the Epitomics (Epit) monoclonal anti-*N*-terminal Myc antibody, 2303 (FDR=6.82%) Myc binding regions were identified within 2377 genes. The number of Myc bound genes slightly exceeded the number of Myc binding regions. This was partly due to Myc’s preference to bind to bidirectional promoters (*i.e.*, to two genes on opposite strands but with closely located transcriptional start sites), and partly due to the -10kb to +5kb promoter regions of two genes that might overlap.

Intersecting the two lists identified 1327 common genes bound by Myc in both data sets. Among them, 1190 had gene expression measures for all microarrays (including exon arrays, Human U133 Plus 2.0, and Agilent arrays). Comparing the high to low Myc conditions identified 445 (37.4% out of 1190) genes up-regulated and 189 (15.9% out of 1190) genes down-regulated in all four P493-6 gene expression data sets, including the NRO, total RNA and all RNA exon array data, as well as total RNA U133 Plus 2.0 array data. As a comparison, among the total of 12,310 genes that were bound in none of the P493-6 ChIP-chip data sets and that had probes in all gene expression microarrays, 1,473 (12.0% out of 12,310) were up-regulated in all four gene expression data sets, and 1,583 (12.9%) were down-regulated in all four data sets. Thus Myc-bound genes are 3.13 (37.4% versus 12.0%) times more likely to be up-regulated than genes not bound by Myc. However, the genes bound by Myc and those not bound by Myc had about the same tendency to be repressed (15.9% versus 12.9% = 1.24). We conclude the most common effect of Myc binding on direct target genes is to activate rather than repress transcription.

**Myc Targets in Human H9 ES Cells**

The promoter ChIP-chip analysis identified 1787 (FDR=7.16%) Myc binding regions in human H9 ES cells, representing 1984 genes. Among these genes, 1806 had gene expression measures for all microarrays (including exon arrays, Human U133 Plus 2.0, and Agilent arrays). 265 (14.7%) out of the 1806 Myc bound genes were elevated in H9 hES cells compared to trophoblasts, and 102 (5.6%) were repressed. As a comparison, among the 13,697 genes not bound by Myc in the H9 hES cells, 1258 (9.2% out of 13,697) were up-regulated in the hES cells, and 1259 (9.2%) were down-regulated. The Myc bound genes were 1.60 (14.7% versus 9.2%) times more likely to be up-regulated in hES cells compared to genes not bound by Myc, and the Myc bound genes were less likely to be repressed (5.6% versus 9.2% = 0.61). Again, this supports the fact that direct targets of Myc binding are preferentially activated rather than repressed.

**Common Myc Targets in Human P493-6 B Cells and H9 ES Cells**

Since the data suggested that the principal effect of Myc binding on its direct targets is to activate transcription, all subsequent analyses were focused on genes elevated in high Myc conditions. Comparing the P493-6 B cell data with the H9 hESC data identified 749 genes that were bound by Myc in both B cells and hES cells. Among these genes, 668 had probes in all gene expression arrays. 80 (12.0%) of the 668 genes were up-regulated in high Myc conditions (*i.e.*, in ES cells, or in P493-6 B cells following Myc induction). These 80 genes were defined as the human B cell and ES cell common Myc targets. As a comparison, among the 11,635 genes that were not bound by Myc in any ChIP-chip data sets and that had probes in all gene expression microarrays and promoter tiling arrays, 168 (1.4%) were up-regulated in high Myc conditions. Therefore, genes bound by Myc in both B cells and hES cells were 8.3 (12.0% versus 1.4%) times more likely to be up-regulated than the genes not bound by Myc.

**Human Core Set of Myc Targets**

Myc binding regions identified by ChIP-seq for the three human cancer cell lines HeLa-S3, HepG2 and K562 were collected from the ENCODE website. For HeLa-S3 and K562 cells the ChIP-seq data were from two different labs (University of Texas (UT) and Yale). For HepG2, ChIP-seq data were generated only by the UT group. Thus, we assessed five ChIP-seq data sets representing three different cancer cell lines. Among the 668 genes bound by Myc in both B cells and hES cells, 501 were bound in all five ENCODE data sets. 73 (14.6%) out of the 501 genes were up-regulated in high Myc conditions, representing a 10.1 (14.6% versus 1.4%) fold enrichment of Myc-bound genes that were up-regulated compared to up-regulated genes not bound by Myc. These 73 genes were defined as the human core set of Myc target genes.

**Species-independent Core Set of Myc Targets**

Two ChIP-chip (anti-Myc-ChIP and biotin-mediated anti-Myc ChIP [11]) and one ChIP-seq [5] data sets for Myc binding in mouse ES cells were collected and analyzed. Mouse genes were matched with their homolog genes in human using HomoloGene (<http://www.ncbi.nlm.nih.gov/sites/entrez?db=homologene>). Among the 501 genes bound by Myc in human B cells, hES cells, and all five ENCODE data sets, 304 had mouse homologs that were bound by Myc in at least two out of the three mouse ES ChIP-chip/ChIP-seq data sets. 51 (16.8%) out of the 304 genes were elevated in high Myc conditions, representing a 11.6 (16.8% versus 1.4%) fold enrichment of Myc-bound up-regulated genes compared to other genes. These 51 genes were defined as species-independent core set of Myc target genes.

**Human Cell Type Restricted Myc Targets**

Comparing the ChIP-chip and gene expression data between P493-6 B cells and H9 ES cells allowed us to define human cell type restricted Myc target genes. For each gene, we defined *C_B_* as the maximal log2 ChIP/control fold change in the P493-6 B cell peaks associated with that gene (averaged across two antibodies). We defined *C_ES_* as the maximal log2 ChIP/control fold change in the H9 ES cell peaks, and *E_B_* to be the log2 genes expression fold change between the high versus low Myc conditions in P493-6 B cells. *E_ES_* was defined as the log2 genes expression fold change between the H9 ES cells and trophoblast cells.

A Myc target gene was defined to be a human B cell specific positive target if all of the following six conditions were satisfied: (1) the gene was bound by Myc in P493-6 B cells but not in H9 ES cells; (2) *C_B_* – *C_ES_* > log2 (1.5); (3) the gene was significantly up-regulated in the high Myc conditions in B cells (in all four B cell expression data sets) but not significantly up-regulated in ES cells; (4) *E_B_* > log2(1.5); (5) *E_ES_* < log2(1.5); and (6) *E_B_* – *E_ES_* > log2(1.5). Here, the binding or differential expression in condition 1 and 3 were determined by statistical significance cutoffs described in the Supplemental Methods sections “ChIP-chip and ChIP-seq Data Analysis” and “Gene Expression Microarray and Exon Array Data Analysis”. Conditions 2, 4, 5 and 6 are additional criteria to ensure that the cell type specificity was not due to cutoff effects, that is, to avoid calling a gene cell type restricted if its binding and/or differential expression was just below the statistical cutoff in one cell type and just above the cutoff in another cell type.

A Myc target gene was defined to be a human B cell-specific Myc repression target if the following six conditions were met: (1) the gene was bound by Myc in P493-6 B cells but not in H9 ES cells; (2) *C_B_* – *C_ES_* > log2 (1.5); (3) the gene was significantly down-regulated in the high Myc conditions in B cells (in all four B cell expression data sets) but not significantly down-regulated in ES cells; (4) –*E_B_* > log2(1.5); (5) –*E_ES_* < log2(1.5); and (6) –*E_B_* + *E_ES_* > log2(1.5).

A Myc target gene was defined to be a human ES cell-specific, Myc induced target if: (1) the gene was bound by Myc in H9 ES cells but not in P493-6 B cells; (2) *C_ES_* – *C_B_* > log2 (1.5); (3) the gene was significantly up-regulated in the ES cells but not in the high Myc conditions in B cells (in all four B cell expression data sets); (4) *E_ES_* > log2(1.5); (5) *E_B_* < log2(1.5); and (6) *E_ES_* – *E_B_* > log2(1.5).

Finally, a Myc target gene was defined to be a human ES cell-specific Myc repression target if (1) the gene was bound by Myc in H9 ES cells but not in P493-6 B cells; (2) *C_ES_* – *C_B_* > log2 (1.5); (3) the gene was significantly down-regulated in the ES cells but not in the high Myc conditions in B cells (in all four B cell expression data sets); (4) –*E_ES_* > log2(1.5); (5) –*E_B_* < log2(1.5); and (6) –*E_ES_* + *E_B_* > log2(1.5).

Using these criteria, we found 21 human B cell-specific Myc activated targets, 29 B cell-specific Myc repression targets, 30 ES cell-specific Myc activated targets, and 22 ES cell-specific Myc repression targets.

**Analysis of Expression Trend of MycTarget Genes in Eμ-*Myc* Mouse**

As described in “Gene Expression Microarray and Exon Array Data Analysis”, microarray data were processed using RMA to compute gene expression levels. There were three sample groups: wild-type (4 samples), pre-malignant Eμ-*Myc* littermates (5 samples), and Eμ-*Myc* lymphoma (13 samples). For each gene, the average expression level for each sample group was computed. A gene is said to have increasing expression levels if its average expression levels satisfy wild-type < Eμ-*Myc* littermates < Eμ-*Myc* lymphoma. A gene is said to have decreasing expression levels if its average expression levels satisfy wild-type > Eμ-*Myc* littermates > Eμ-*Myc* lymphoma.

**Gene Set Enrichment Analysis of genes enriched in premalignant Eμ-*Myc* B220+ B cells compared to their wild type counterparts**

RMA derived gene expression values were imported into GSEA v2.06 software (<http://www.broadinstitute.org/gsea/index.jsp>), and premalignant Eμ-*Myc* B220+ samples were analyzed compared to wild type B220+ samples based on GSEA default parameter settings (Permutation type = gene set). Data are shown in **Supplemental Figure S2**.

**Analysis of 8129 GEO Microarray Samples**

8129 Affymetrix Human U133A microarray samples were collected from GEO [12,13]. The samples were normalized and processed using fRMA [14] to compute gene expression levels. For each sample, we computed the MCS score as the average log2 gene expression across all genes in the 51 core Myc target gene set. The correlation coefficient between the MCS score and the log2 Myc expression level was 0.464. By randomly choosing 51 genes from the array 10000 times, we estimate the *p* value of the MCS-Myc correlation to be p < 0.001. We define high Myc and high MCS samples to be microarray samples whose *MYC* expression and MCS score are both above the 90^th^ percentiles across all samples. For each cell or tissue type, we counted how many samples of that type exist in all 8129 microarray samples and how many samples of that type have high *Myc* and high MCS expression. We then conduct a one-sided Fisher's exact test for each cell or tissue type with a FDR value cutoff of 0.05 to identify which type are enriched in high *Myc* and high MCS while accounting for the given distribution of cell (Ioba are spontaneously immortalized human conjunctival epithelial cell lines; M07e is an acute megakaryoblastic leukemia cell line; K562 is a cell line derived from chronic myelocytic leukemia) and tissues types in the data (**Figure 7C**). Notably, we find a significant enrichment of tumors in high *Myc* and high MCS samples, 236 of 246 samples, compared to the number of tumors in all samples, 4690 of 8129 samples (**Figure 7D**).

**supplemental References**
